# Patterns of percutaneous transthoracic needle biopsy (PTNB) of the lung and risk of PTNB-related severe pneumothorax: A nationwide population-based study

**DOI:** 10.1371/journal.pone.0235599

**Published:** 2020-07-10

**Authors:** Bo Ram Yang, Mi-Sook Kim, Chang Min Park, Soon Ho Yoon, Kum Ju Chae, Joongyub Lee

**Affiliations:** 1 Medical Research Collaborating Center, Seoul National University Hospital, Seoul, Republic of Korea; 2 Department of Radiology, Seoul National University College of Medicine, Seoul, Republic of Korea; 3 Institute of Radiation Medicine, Seoul National University Medical Research Center, Seoul, Republic of Korea; 4 Department of Radiology, Research Institute of Clinical Medicine of Jeonbuk National University-Biomedical Research Institute of Jeonbuk National University Hospital, Jeonju, Republic of Korea; 5 School of Medicine, Inha University, Incheon, Republic of Korea; 6 Department of Prevention and Management, Inha University Hospital, Incheon, Republic of Korea; Vanderbilt University Medical Center, UNITED STATES

## Abstract

**Background:**

As percutaneous transthoracic needle biopsy (PTNB) of the lung is a well-established diagnostic method for the evaluating pulmonary lesions, evidence of safety based on representative data is limited. This study investigated the practice patterns of PTNB of the lung and assessed the incidence and risk factors of PTNB-related severe pneumothorax in Korea.

**Methods:**

We used a national-level health insurance database between January 1, 2007 and December 31, 2015. Patients who underwent PTNB of the lung were identified using procedure codes for organ biopsy, fluoroscopy, computed tomography, chest radiography, and lung-related diagnosis codes. The annual age-/sex-standardized rate of PTNB and the incidence of PTNB-related severe pneumothorax were calculated. We defined severe pneumothorax as the pneumothorax requiring intervention. The odds ratios of risk factors were assessed by a generalized estimating equation model with exchangeable working correlation matrix to address clustering effect within institution.

**Results:**

A total of 66,754 patients were identified between 2007 and 2015. The annual age-/sex-standardized rate of PTNB per 100,000 population was 19.6 in 2007 and 22.4 in 2015, and it showed an increasing trend. The incidence of severe pneumothorax was 2.4% overall: 2.5% in men and 1.2% in women, and 2.6%, 2.7%, 2.1%, 2.1%, 1.9%, 2.4%, and 2.4% from 2009 to 2015. Older age (≥60), male sex, presence of chronic obstructive pulmonary disease, receiving treatment in an urban or rural area versus a metropolitan area, and receiving treatment at a general hospital were significantly associated with the risk of severe pneumothorax.

**Conclusions:**

Considering the increasing trend of PTNB, more attention needs to be paid to patients with risk factors for severe pneumothorax.

## Introduction

Percutaneous transthoracic needle biopsy (PTNB) of the lung is a well-established diagnostic method for the pathologic evaluation of pulmonary lesions. It has been proven to be minimally invasive and effective, and is therefore recommended by the relevant guidelines in cases of peripheral lung cancer [1, 2]. For lung cancer, a diagnostic accuracy of almost 90% for malignancies was reported, as well as a diagnostic accuracy of 80% for benign lesions [3–6]. Lung cancer is the leading cause of cancer death [7, 8], and the number of incident cases of lung cancer has shown an increasing trend in Korea [9]. It is assumed that many PTNBs of the lung are performed, but the extent and characteristics of PTNBs have not been assessed on a nationwide scale.

Even though PTNB is considered to be relatively safe procedure, pneumothorax, pulmonary venous air embolism, hemoptysis, and death have been reported as complications related to PTNB [10, 11]. Pneumothorax is the most common PTNB-related complication. Since PTNB is a diagnostic procedure, the acceptable potential risk should be considered more restrictively than is the case for therapeutic or curative procedures. Evidence of safety based on an epidemiologic study is therefore needed.

The published rates of pneumothorax as a complication of PTNB vary widely according to the study setting, and the majority of previous studies included fewer than 1,000 patients and were conducted using records from a single institution [12–15]. As it is important to investigate this issue on a national level, several studies on the complications of PTNB have been performed in the United States (US) and the United Kingdom [11, 16, 17]; however, a representative study of an Asian population remains a desideratum.

Therefore, the purpose of this study was to evaluate the rate of PTNB, the incidence of severe pneumothorax as a complication of PTNB, and the risk factors for PTNB-related severe pneumothorax in the Korean population using a national-level health insurance database.

## Materials and methods

### Data sources

The Korean National Health Insurance system was launched in 1977 and achieved universal coverage of the entire Korean population in 1989. Health insurance claims data, also known as Health Insurance and Review Assessment (HIRA) data, are publicly available for research after de-identification. The HIRA data are comprehensive, and contain general information (beneficiary ID, age, sex, type of insurance, indicators for inpatient/outpatient care, medical institution ID, type of medical institution, dates, etc.), healthcare services (procedures, inpatient prescriptions, operations, examinations, etc.), diagnoses, and outpatient prescriptions. Diagnoses were coded using the Korean Standard Classification of Diseases Version 6, which is based on International Statistical Classification of Diseases 10th revision (ICD-10) codes. [18]

We obtained claims data for patients who underwent deep-organ needle biopsy from January 1, 2007 to December 31, 2015.

### Selection of the study population

As the HIRA procedure codes do not contain an exclusive code for PTNB, but only a code for deep-organ needle biopsy, which includes PTNB, we defined PTNB using the HIRA procedure codes for the imaging methods that accompanied PTNB, the type of medical institution, specialty, and diagnosis codes. The patients were defined as having undergone PTNB when the following eligibility criteria were met on the same date: 1) deep-organ needle biopsy; 2) chest computed tomography (CT) or diagnostic fluoroscopy guidance; 3) chest radiography; 4) admission to a tertiary medical institution or outpatient visit at a Department of Thoracic and Cardiovascular Surgery or Pulmonology at a tertiary medical institution; and 5) a diagnostic code involving lung-related disease. Lung-related diseases were selected based on Wiener et al. [17], and amended after expert review. Abnormal findings on diagnostic imaging of lung (ICD-10: R91), malignant neoplasm of trachea (C33), malignant neoplasm of bronchus and lung (C34), secondary malignant neoplasm of lung (C78.0), benign neoplasm of bronchus and lung (D14.3), neoplasm of uncertain behavior of trachea, bronchus and lung (D38.1), and neoplasm of unspecified behavior of respiratory system (D49.1) were included.

For the analysis of complications among patients who underwent PTNB, we excluded patients who underwent PTNB between January 1, 2007 and December 31, 2008 in order to restrict the sample to new cases of PTNB using a 2-year pre-index period, and we also excluded those who were diagnosed with a disease related to the complication before the date of PTNB in order to identify incident cases of complications.

### Complications of PTNBs and covariates

Cases involving a complication of PTNB were defined as pneumothorax (ICD-10: J93) requiring thoracostomy or percutaneous catheter drainage, using the corresponding HIRA procedure codes on the same date as the PTNB.

The covariates that were analyzed included age group (20–49, 50–59, 60–69, 70–79, over 80), sex, insurance status (health insurance, Medical Aid, Veterans Health Service), imaging guidance (CT guidance or fluoroscopy guidance), year of PTNB, comorbidities (chronic obstructive pulmonary disease, pleural effusion), Charlson Comorbidity Index (CCI), and history of lung surgery during the 1-year period before the PTNB date. The CCI score was calculated using the method proposed by Quan et al. [19]. The characteristics of medical institutions included the type of medical institution (superior general hospital, general hospital) and region (Seoul metropolitan area, urban area, rural area). The type of medical institution was classified as follows: (1) general hospitals were defined as medical institutions with at least seven departments and 100–299 beds, or nine departments and more than 300 beds; and (2) superior general hospitals were defined as medical institutions with more than 20 departments that had been designated by the Medical Service Act (Act No. 9386) of the Ministry of Health and Welfare of South Korea on the basis of their educational role, manpower, facilities, and equipment [20]. The Seoul metropolitan area consisted of Seoul metropolitan city, Incheon metropolitan city, and Gyeonggi Province; urban areas consisted of other metropolitan cities (Daejeon, Daegu, Gwangju, Busan, Ulsan); and the remaining regions were defined as rural areas.

### Statistical analysis

Baseline characteristics were described using proportions for categorical variables. The age-and sex-specific biopsy rate per 100,000 population was calculated by dividing the number of patients who underwent PTNB each year by the number of beneficiaries of Korean national health insurance in that year and multiplying the result by 100,000. Data from the Korea National Census conducted in 2010 were used to define the standard population. Direct standardization was used to calculate the annual age- and sex-standardized biopsy rate, with 95% confidence intervals (CIs) estimated based on the Poisson distribution. The age-standardized rate was calculated for sex-specific populations, and annual trends were tested using a regression model [21]

Considering the possibility that the patients were clustered within medical institutions, the incidence rate (%) of complications was estimated using the generalized estimating equation (GEE) method, assuming an exchangeable correlation structure for analyzing the correlated data, and the 95% CI of estimates was calculated using empirical standard errors [22]. The incidence rate was stratified by age group, insurance status, type of imaging guidance, year of PTNB, presence of comorbidities, lung surgery, CCI score, region, and type of medical care institution. Sex-specific incidence rates were also calculated.

Association between risk of severe pneumothorax and characteristics of patients and hospital were performed using GEE model. We applied a GEE to account for correlation within institutions, assuming the binomial family, a logit link function, and an exchangeable correlation structure. The odds ratios (ORs) and 95% CIs of severe pneumothorax associated with age, sex, insurance status, imaging guidance, type of medical institution, region, presence of chronic obstructive pulmonary disease, pleural effusion, lung surgery and CCI score were calculated in univariable analysis. After forward selection method was used, the multivariable models included age, sex, imaging guidance, type of medical institution, region, presence of chronic obstructive pulmonary disease, and CCI score.

All statistical analyses were conducted using SAS Enterprise Guide, version 6.1 (SAS Institute, Inc., Cary, NC, USA).

### Ethical statement

The present study was exempted from review by the Institutional Review Board of Seoul National University Hospital (IRB number: E-1604-054-753).

## Results

**Fig 1:**
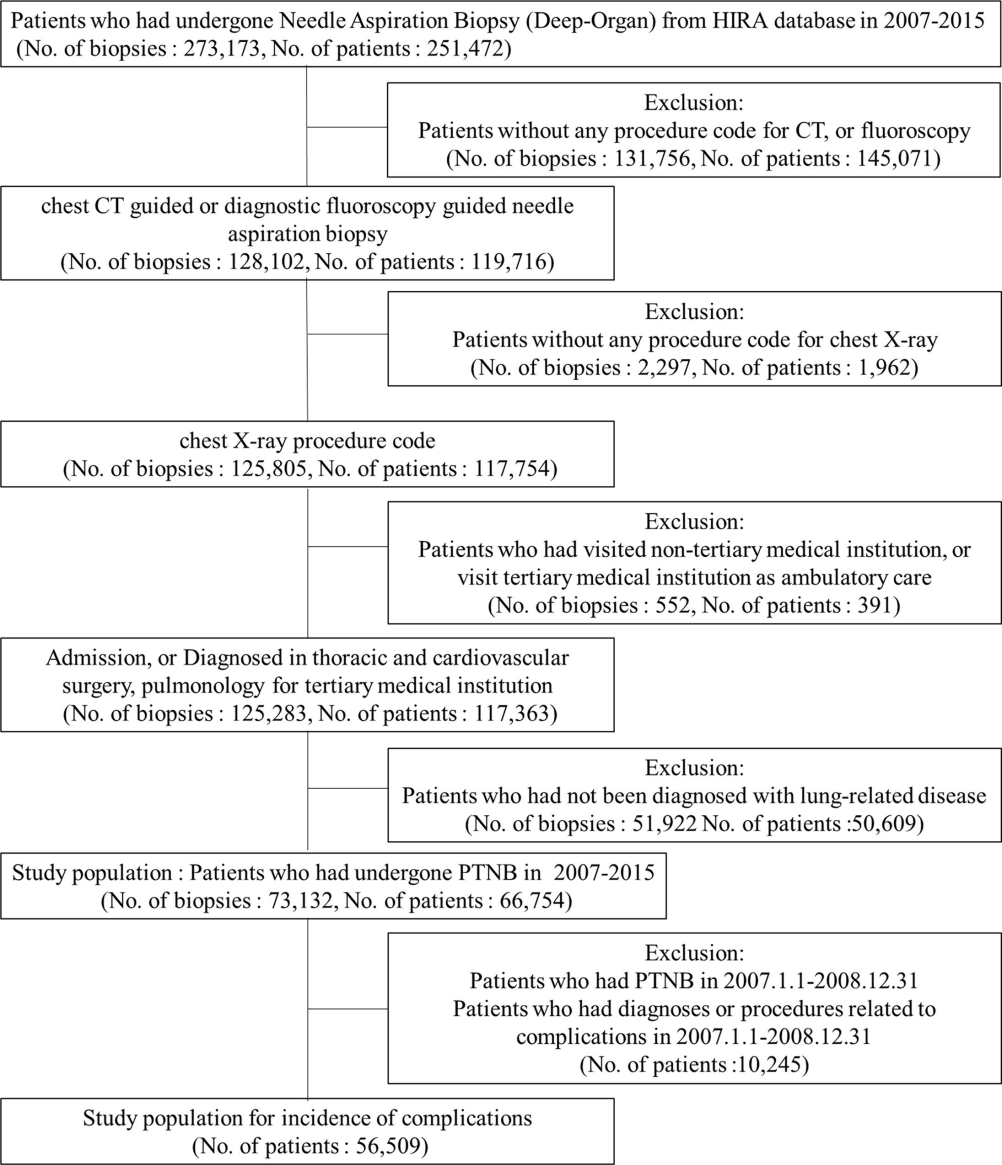
Flowchart of the study population.

From the HIRA database, we identified 73,132 biopsies of 66,754 patients who met our criteria for PTNB of the lung from 2007 to 2015, and among them, 55,687 patients were included in the analysis of complications (Fig 1).

The baseline characteristics of patients who underwent PTNB of the lung from 2007 to 2015 are shown in Table 1. Among the 73,132 biopsies, 65.5% were performed in men, and the proportions of patients in their 60s and 70s were over 30%. The proportion of CT-guided PTNB was higher than that of fluoroscopy-guided PTNB (Table 1).

**Table 1:** Baseline characteristics of percutaneous transthoracic needle biopsies of the lung from 2007 to 2015.

Characteristics	Total		Male		Female	
	N	(%)	N	(%)	N	(%)
**Total**	73,132	(100.0)	47,936	(65.5)	25,196	(34.5)
**Age group**						
20–49	6,356	(8.7)	3,258	(6.8)	3,098	(12.3)
50–59	13,261	(18.1)	7,866	(16.4)	5,395	(21.4)
60–69	21,997	(30.1)	15,149	(31.6)	6,848	(27.2)
70–79	25,136	(34.4)	17,539	(36.6)	7,597	(30.2)
80–89	6,185	(8.5)	4,014	(8.4)	2,171	(8.6)
90+	197	(0.3)	110	(0.2)	87	(0.3)
**Insurance status**						
Health insurance	68,326	(93.4)	45,078	(94.0)	23,248	(92.3)
Medical Aid	4,629	(6.3)	2,681	(5.6)	1,948	(7.7)
Veterans Health Service	177	(0.2)	177	(0.4)	0	(0.0)
**Guidance**^a^						
Computed tomography guidance	47,673	(65.2)	31,372	(65.4)	16,301	(64.7)
Fluoroscopy guidance	33,337	(45.6)	21,800	(45.5)	11,537	(45.8)
**Year**						
2007	6,054	(8.3)	3,991	(8.3)	2,063	(8.2)
2008	6,354	(8.7)	4,255	(8.9)	2,099	(8.3)
2009	6,931	(9.5)	4,621	(9.6)	2,310	(9.2)
2010	7,314	(10.0)	4,878	(10.2)	2,436	(9.7)
2011	8,545	(11.7)	5,588	(11.7)	2,957	(11.7)
2012	9,287	(12.7)	5,974	(12.5)	3,313	(13.1)
2013	9,176	(12.5)	5,938	(12.4)	3,238	(12.9)
2014	9,646	(13.2)	6,252	(13.0)	3,394	(13.5)
2015	9,825	(13.4)	6,439	(13.4)	3,386	(13.4)
**Medical institution**						
Superior general hospital	50,138	(68.6)	32,602	(68.0)	17,536	(69.6)
General hospital	22,994	(31.4)	15,334	(32.0)	7,660	(30.4)
**Region**						
Seoul metropolitan area	46,066	(63.0)	29,360	(61.2)	16,706	(66.3)
Urban area	15,001	(20.5)	10,168	(21.2)	4,833	(19.2)
Rural area	12,065	(16.5)	8,408	(17.5)	3,657	(14.5)

^a^ The categories of type of guidance were not mutually exclusive.

**Fig 2:**
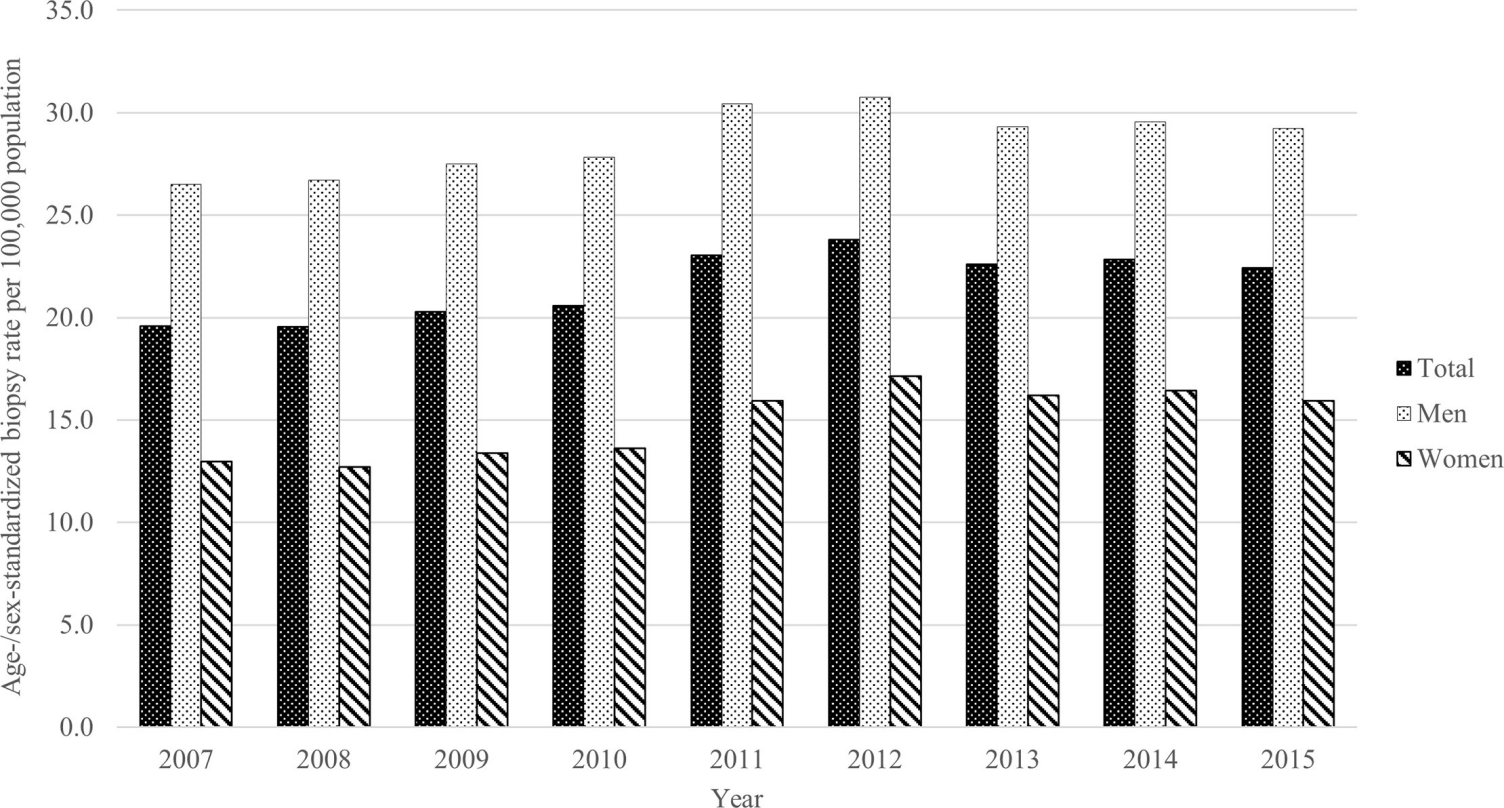
Age-/sex-standardized percutaneous transthoracic needle biopsy rates among the Korean population.

The crude biopsy rate was 16.7 per 100,000 population in 2007, and 24.3 per 100,000 population in 2015. The age-/sex-standardized rates were 19.6 per 100,000 population in 2007 and 22.4 per 100,000 in 2015, with a peak in 2012. The age-standardized rate in 2015 was 29.2 per 100,000 in men and 15.9 per 100,000 in women. The biopsy rate showed a significantly increasing trend (p = 0.008), both in men (p = 0.019) and women (p = 0.0039) (Fig 2).

The incidence rate of severe pneumothorax was 2.4% (95% CI: 2.15%-2.73%) among patients who newly underwent PTNB during 2007–2015, with a breakdown by sex of 2.9% (95% CI: 2.55%-3.21%) for men, and 1.4% (95% CI: 1.14%-1.69%) for women. The incidence rate of severe pneumothorax increased by age group, ranging from 1.6% in those aged 20–49 to 2.6% in the 80+ age group. The incidence of PTNB-related severe pneumothorax was 2.5% in CT-guided PTNBs, in contrast to 2.1% in fluoroscopy-guided PTNBs. The incidence rate was lower in the Seoul metropolitan area (2.1%, 95% CI: 1.74%-2.40%) than in urban areas (3.3%, 95% CI: 2.70%-3.93%) and rural areas (2.3%, 95% CI: 1.79%-2.86%), and it was lower in superior general hospitals (2.0%, 95% CI: 1.65%-2.38%) than in general hospitals (2.7%, 95% CI: 2.33%-3.10%) (Table 2).

**Table 2:** Incidence rate of severe pneumothorax among patients who underwent percutaneous transthoracic needle biopsy of the lung.

Characteristics	Total	No. of events	IR % (95% CI)	Men	No. of events	IR % (95% CI)	Women	No. of events	IR % (95% CI)
**Total**	56,509	1,145	2.4 (2.15, 2.73)	36,957	907	2.9 (2.55, 3.21)	19,552	238	1.4 (1.14, 1.69)
**Age group**									
20–49	4,589	54	1.6 (1.11, 2.06)	2,321	26	1.5 (0.87, 2.17)	2,268	28	1.4 (0.88, 2.01)
50–59	10,170	142	1.6 (1.23, 1.93)	5,979	102	1.9 (1.48, 2.41)	4,191	40	1.0 (0.66, 1.25)
60–69	16,615	329	2.4 (2.02, 2.82)	11,450	276	2.9 (2.39, 3.33)	5,165	53	1.1 (0.75, 1.54)
70–79	19,741	485	2.7 (2.31, 3.02)	13,751	402	3.1 (2.68, 3.53)	5,990	83	1.5 (1.11, 1.92)
80+	5,394	135	2.6 (2.05, 3.19)	3,456	101	3.1 (2.40, 3.89)	1,938	34	1.8 (1.09, 2.44)
**Insurance status**									
Health insurance	52,876	1,050	2.4 (2.13, 2.71)	34,789	838	2.8 (2.51, 3.17)	18,087	212	1.4 (1.1, 1.67)
Medical Aid, VHS	3,633	95	2.6 (2.03, 3.18)	2,168	69	3.2 (2.37, 4.02)	1,465	26	1.7 (1.13, 2.36)
**Guidance**^a^									
CT guidance	31,589	673	2.5 (2.13, 2.86)	20683	531	2.9 (2.48, 3.29)	10906	142	1.5 (1.09, 1.91)
Fluoroscopy guidance	18,724	331	2.1 (1.68, 2.50)	12,175	264	2.6 (2.02, 3.1)	6,549	67	1.2 (0.79, 1.59)
**Year**									
2009	6,545	152	2.6 (2.04, 3.23)	4,350	116	2.9 (2.19, 3.56)	2,195	36	2.0 (1.12, 2.78)
2010	6,910	175	2.7 (2.13, 3.36)	4,611	146	3.4 (2.62, 4.09)	2,299	29	1.3 (0.76, 1.89)
2011	7,956	149	2.1 (1.61, 2.57)	5,205	122	2.6 (1.97, 3.14)	2,751	27	1.1 (0.61, 1.53)
2012	8,631	153	2.1 (1.63, 2.51)	5,556	123	2.5 (1.96, 3.05)	3,075	30	1.0 (0.63, 1.39)
2013	8,557	146	1.9 (1.51, 2.22)	5,543	108	2.1 (1.68, 2.61)	3,014	38	1.3 (0.84, 1.74)
2014	8,912	194	2.4 (1.86, 2.85)	5,801	157	2.9 (2.30, 3.49)	3,111	37	1.3 (0.75, 1.76)
2015	8,998	176	2.4 (1.87, 2.95)	5,891	135	2.7 (2.07, 3.37)	3,107	41	1.6 (0.93, 2.21)
**Comorbidities**									
COPD	26,889	647	2.9 (2.53, 3.23)	18,828	534	3.4 (2.96, 3.79)	8,061	113	1.5 (1.17, 1.91)
PE	2,765	51	2.0 (1.38, 2.59)	1,801	39	2.4 (1.57, 3.18)	964	12	1.2 (0.57, 1.90)
**CCI score**									
0	11,734	216	2.1 (1.68, 2.46)	7,162	166	2.5 (2.05, 3.02)	4,572	50	1.2 (0.77, 1.58)
1	11,200	242	2.3 (1.92, 2.75)	7,286	203	2.9 (2.41, 3.47)	3,914	39	1.1 (0.69, 1.45)
2	10,814	225	2.6 (2.12, 3.06)	6,919	170	2.9 (2.39, 3.48)	3,895	55	1.7 (1.14, 2.19)
3+	22,761	462	2.3 (2.00, 2.69)	15,590	368	2.7 (2.31, 3.09)	7,171	94	1.4 (1.03, 1.83)
**Lung surgery**									
Yes	1,113	14	1.6 (0.63, 2.54)	804	8	1.2 (0.28, 2.11)	309	6	2.2 (0.11, 4.38)
**Region**									
Seoul metropolitan area	35,272	522	2.1 (1.74, 2.40)	22,559	409	2.4 (2.01, 2.70)	12,713	113	1.2 (0.86, 1.54)
Urban area	11,843	409	3.3 (2.70, 3.93)	7,898	323	4.0 (3.29, 4.71)	3,945	86	1.9 (1.35, 2.49)
Rural area	9,394	214	2.3 (1.79, 2.86)	6,500	175	2.8 (2.13, 3.45)	2,894	39	1.3 (0.83, 1.82)
**Medical care institution**									
Superior general hospital	38,775	716	2.0 (1.65, 2.38)	25,208	571	2.5 (2.03, 2.90)	13,567	145	1.2 (0.82, 1.53)
General hospital	17,734	429	2.7 (2.33, 3.10)	11,749	336	3.2 (2.69, 3.64)	5,985	93	1.7 (1.30, 2.13)

IR: incidence rate, VHS: Veterans Health Service, CT: computed tomography, COPD: chronic obstructive pulmonary disease, PE: pleural effusion, CCI: Charlson Comorbidity Index.

^a^ The categories of type of guidance were not mutually exclusive.

After adjustment, significant ORs for severe pneumothorax were found for sex, age group, the presence of COPD, region, and type of medical care institution. The adjusted OR (aOR) for severe pneumothorax was 1.84 (95% CI: 1.61–2.11) in men compared to women. Patients in their 60s, 70s, and those over 80 showed an increased risk (aOR: 1.39, 95% CI: 1.03–1.88, aOR: 1.69, 95% CI: 1.22–2.33, aOR: 1.68, 95% CI: 1.17–2.42, respectively) compared with patients <50 years. The presence of COPD (aOR: 1.35, 95% CI: 1.18–1.54), residence in an urban region (aOR: 1.59, 95% CI: 1.21–2.10), and receiving treatment at a general hospital (aOR: 1.39, 95% CI: 1.13–1.71) were linked with an increased risk of severe pneumothorax (Table 3).

**Table 3:** Risk of severe pneumothorax associated with patients’ characteristics.

General characteristics	Total	No. of events	OR	(95% CI)		Adjusted OR	(95% CI)	
**Sex**								
Women	19,552	238	Ref			Ref		
Men	36,957	907	1.84	(1.64,	2.05)	1.84	(1.61,	2.11)
**Age group**								
20–49	4,589	54	Ref			Ref		
50–59	10,170	142	1.13	(0.88,	1.43)	1.05	(0.76,	1.47)
60–69	16,615	329	1.50	(1.21,	1.87)	1.39	(1.03,	1.88)
70–79	19,741	485	1.75	(1.37,	2.23)	1.69	(1.22,	2.33)
80+	5,394	135	1.76	(1.33,	2.33)	1.68	(1.17,	2.42)
**Insurance status**								
Health insurance	52,876	1,050	Ref					
Medical Aid, Veterans Health Service	3,633	95	1.15	(0.95,	1.40)			
**Guidance**^a^								
Fluoroscopy guidance	18,724	331	Ref			Ref		
CT guidance	31,589	673	1.21	(0.96,	1.54)	1.19	(0.95,	1.50)
**Comorbidities**								
COPD	26,889	647	1.38	(1.26,	1.51)	1.35	(1.18,	1.54)
Pleural effusion	2,765	51	0.88	(0.69,	1.11)			
**CCI score**								
0	11,734	216	Ref			Ref		
1	11,200	242	1.14	(0.98,	1.33)	0.95	(0.79,	1.15)
2	10,814	225	1.15	(0.96,	1.39)	0.89	(0.71,	1.12)
3+	22,761	462	1.13	(0.99,	1.29)	0.84	(0.70,	1.01)
**Lung surgery**								
Lung surgery	1,113	14	0.74	(0.49,	1.13)			
**Region**								
Seoul metropolitan area	35,272	522	Ref					
Urban area	11,843	409	1.64	(1.28,	2.11)	1.59	(1.21,	2.10)
Rural area	9,394	214	1.14	(0.86,	1.53)	1.06	(0.79,	1.43)
**Medical care institution**								
Superior general hospital	38,775	716	Ref					
General hospital	17,734	429	1.40	(1.13,	1.74)	1.39	(1.13,	1.71)

OR: odds ratio, CI: confidence interval, COPD: chronic obstructive pulmonary disease, CCI: Charlson Comorbidity Index.

^a^ The patients with both types of guidance were excluded.

## Discussion

Our study evaluated the annual rate of PTNB, the rate of severe pneumothorax as a complication, and factors related to the incidence of PTNB-related severe pneumothorax in Korea using a nationally representative database. The age-/sex-standardized rates showed an increasing trend from 2007 to 2015, both in men and women. The rate of severe pneumothorax as a complication was 2.4%. The risk factors of PTNB-related severe pneumothorax were male sex, older age, the presence of COPD, residence in urban and rural regions, and type of hospital.

The increasing trend of PTNB was interpreted as resulting from increasing rates of participation in opportunistic cancer screening programs. Although screening for lung cancer was not included in the organized cancer screening program implemented by the Korean government, in recent years, most general hospitals have provided cancer screening programs. The number of patients participating in opportunistic cancer screening programs has increased, despite their relatively high cost [23]. Furthermore, the annual trends in the frequency and age-/sex-standardized rate of PTNB procedures were similar to trends in lung cancer surgery incidence in Korea, which showed an increasing trend between 2010 and 2014, both in men and women [24]. The number of repeated biopsies could have partially contributed to the increasing trend. According to a survey of Korean radiologists in 2016, the number of repeated biopsies increased over the past 5 years, and molecular analysis for an established target therapy and clinical trials of new drugs were identified as the main reasons [25].

The complication rate in the current study was in line with the results of previous studies. In previous studies, post-biopsy severe pneumothorax requiring intervention occurred in 1%-14.2% of patients [12, 17, 26–28]. In a cross-sectional study conducted in 4 states in the US using state-level inpatient and ambulatory surgery databases that included 15,865 patients, the rate of pneumothorax requiring a chest tube was 6.6% (95% CI: 6.0%-7.2%) of all biopsies [17]. According to a meta-analysis of 49 articles on CT-guided transthoracic lung biopsy, the pooled complication rates of pneumothorax requiring intervention were 5.6% for core biopsies and 4.3% for fine-needle biopsies [29]. The complication rates also varied in studies conducted in Asia. The chest tube insertion rate was 1% of 660 procedures in Taiwanese patients reported by Yeow et al., while it was 14.2% of 289 patients in a study in Japan conducted by Saji et al. [12, 28].

Older age was identified as a risk factor for complications of PTNB. As the age of patients increased, the incidence of complications increased. This could be explained by age-related physiological changes, such as weakening of the elastic recoil of lung parenchyma. Similarly, Geraghty et al. reported that being more than 60 years old was a risk factor for pneumothorax after transthoracic needle biopsy, and the risk remained when old age was defined as 70 or older [13]. In single-center studies, older patient age was found to be an independent risk factor for pneumothorax [14, 30]. However, some studies showed that the risk peaked in patients in their 60s or 70s [17, 31]. These results could be explained by the possibility that relatively few patients who were very elderly or in an inadequate condition to undergo the procedure were included. This factor is known as healthy-user bias.

The presence of COPD increased the risk of pneumothorax by 1.27 times after adjusting for other covariates, and similar findings were reported in previous studies that identified COPD as a risk factor [15, 17, 32]. According to Anderson et al., the incidence of pneumothorax after PTNB did not differ based on the presence of obstructive lung disease; however, patients with obstructive lung disease more frequently required an intervention with a chest tube when pneumothorax did occur [33]. The association of the presence of COPD with complications was likely influenced by the definition of complications, which was restricted to pneumothorax requiring an intervention.

The complication rate of severe pneumothorax was relatively low at superior general hospitals, and the association between the level of medical institution and the complication rate may be explained by differences in clinicians’ proficiency due to the case volume at larger institutions. According to a notification of the Ministry of Health and Welfare, the treatment of serious diseases that require high-tech treatment such as surgery, as well as treatments requiring the use of multiple facilities and specialized equipment, fall within the scope of superior general hospitals, and centralization among upper-level institutions was also reported for lung cancer surgery [24].

Compared with metropolitan regions, urban regions were associated with an increased risk of PTNB-related severe pneumothorax. A similar trend was found among patients undergoing outpatient transthoracic needle biopsies in the US [16]. Lung cancer is a serious, life-threatening disease, and patients in whom cancer is suspected prefer to receive cutting-edge treatments at medical institutions with experienced and up-to-date facilities, usually located near the city of Seoul. The accessibility of the Seoul metropolitan area has improved due to advances in transportation, such as high-speed trains and airplanes, and it is possible to reach the Seoul area from anywhere in the nation within a day [34]. This study has a number of strengths. Because we used a nationwide health insurance database that covers nearly the entire Korean population, our results are nationally representative. Unlike related studies using databases from a single hospital or several hospitals, we were able to explore relative risk among regions and types of hospitals.

However, the results of our study need to be interpreted cautiously. First, since the HIRA database did not have detailed information on patients’ clinical condition, the accuracy of the diagnoses could not be confirmed. To overcome this limitation, we defined complications using diagnosis and procedure codes that were essential for reimbursement. Another limitation is that we included patient-level factors and characteristics of medical institutions as risk factors for complications, however, only the COPD and pleural effusion were contained as comorbidities, and other comorbidities including emphysema, interstitial lung diseases, and congestive heart failure were not considered. Further studies with comprehensive risk factors would be desirable. Lastly, the characteristics of the target lesions (i.e. location in upper lobe, size, pleural-lesion angle, and depth of approach) and proficiency of operating physicians or radiologists which were relevant factors in risk of pneumothorax could not be evaluated in this study due to the lack of information in the claims database. Furthermore, these information is the basis for judging the appropriateness of modality selection. Transbronchial biopsy during bronchoscopy is preferred for lesions near to bronchial tree, because it does not cross the pleura, transbronchial approach has been reported lower incidence of pneumothorax [35]. However, due to the absence of the information in the claims database, it was difficult to determine whether incidence of pneumothorax was influenced by the selection of modality and lesion characteristics.

## Conclusions

This is the first nationwide study to evaluate the practice patterns of PTNB in Korea and to calculate the complication rate of PTNB-related severe pneumothorax and related factors using a health insurance database that includes the entire Korean population. Considering the increasing trend of PTNB, more attention needs be paid to patients with risk factors for complications of PTNB.
